# Erucin, a Natural Isothiocyanate, Prevents Polyglutamine-Induced Toxicity in *Caenorhabditis elegans* via *aak-2*/AMPK and *daf-16*/FOXO Signaling

**DOI:** 10.3390/ijms252212220

**Published:** 2024-11-14

**Authors:** Martina Balducci, Julia Tortajada Pérez, Cristina Trujillo del Río, Mar Collado Pérez, Andrea del Valle Carranza, Ana Pilar Gomez Escribano, Rafael P. Vázquez-Manrique, Andrea Tarozzi

**Affiliations:** 1 Department for Life Quality Studies, University of Bologna, 47921 Rimini, Italy; 2 Laboratory of Molecular, Cellular and Genomic Biomedicine, Instituto de Investigación Sanitaria La Fe, 46012 Valencia, Spain; julia_tortajada@iislafe.es (J.T.P.); cristina_trujillo@iislafe.es (C.T.d.R.); mar_collado@iislafe.es (M.C.P.); andrea_carranza@iislafe.es (A.d.V.C.); ana_pilar_gomez@iislafe.es (A.P.G.E.); rafael_vazquez@iislafe.es (R.P.V.-M.); 3 Joint Unit for Rare Diseases IIS La Fe-CIPF, 46012 Valencia, Spain; 4 Centro de Investigación Biomédica en Red (CIBER), 28029 Madrid, Spain; 5 Biostructures and Biosystems National Institute (INBB), 00136 Rome, Italy

**Keywords:** erucin, *Caenorhabditis elegans*, neuroprotection, polyQ toxicity, AMPK, *daf-16*/FOXO

## Abstract

Several neurodegenerative diseases (NDDs), such as Huntington’s disease, six of the spinocerebellar ataxias, dentatorubral-pallidoluysian atrophy, and spinobulbar muscular atrophy, are caused by abnormally long polyglutamine (polyQ) tracts. Natural compounds capable of alleviating polyQ-induced toxicity are currently of great interest. In this work, we investigated the modulatory effect against polyQ neurotoxic aggregates exerted by erucin (ERN), an isothiocyanate naturally present in its precursor glucoerucin in rocket salad leaves and in its oxidized form, sulforaphane (SFN), in broccoli. Using *C. elegans* models expressing polyQ in different tissues, we demonstrated that ERN protects against polyQ-induced toxicity and that its action depends on the catalytic subunit of AMP-activated protein kinase (*aak-2*/AMPKα2) and, downstream in this pathway, on the *daf-16*/FOXO transcription factor, since nematodes deficient in *aak-2*/AMPKα2 and *daf-16* did not respond to the treatment, respectively. Although triggered by a different source of neurotoxicity than polyQ diseases, i.e., by α-synuclein (α-syn) aggregates, Parkinson’s disease (PD) was also considered in our study. Our results showed that ERN reduces α-syn aggregates and slightly improves the motility of worms. Therefore, further preclinical studies in mouse models of protein aggregation are justified and could provide insights into testing whether ERN could be a potential neuroprotective compound in humans.

## 1. Introduction

Abnormal expansions of simple sequences in the genome represent a common risk factor for many human diseases, most of which concern the nervous system [1]. Microsatellites may fall in noncoding DNA regions, inducing both loss- and gain-of-function effects through different pathways, which generate potentially aberrant proteins in the latter case. When microsatellite expansions fall within encoding tracts of DNA, they involve trinucleotide repeats, mostly regarding the CAG repeats encoding glutamine, which specifically is the causative factor of nine neurodegenerative diseases (NDDs) [2]. Depending on the poly-glutamine (polyQ) tract lengths and the genes involved, the caused diseases present different natures and phenotypes: Huntington’s disease, six of the spinocerebellar ataxias, dentatorubral-pallidoluysian atrophy, and spinobulbar muscular atrophy (SBMA) are all included in the polyQ expansion diseases [1]. Except for SBMA, which occurs only in men, the other eight polyQ disorders are inherited in an autosomal dominant manner [2]. Moreover, the length of CAG repeats increases through transmission to subsequent generations and is related to an earlier disease onset and severe phenotype [3]. PolyQ tracts imply the formation of neurotoxic proteins that are very prone to losing their folding and forming aggregates [1,2]. In some cases, the loss of protein folding causes the formation of inclusion bodies (IBs), which are composed of mutant proteins with unrelated proteins sequestered within [4]. Therefore, polyQ expansions within endogenous proteins represent the same source of toxicity of various NDDs, probably due to the different expression patterns of the involved genes, which implies malfunctions in different neuronal types [2]. Whatever the neuronal cell nature, all polyQ diseases are characterized by a progressive deterioration of neurons accumulating aggregated proteins within [3].

The fully sequenced genome of the nematode *Caenorhabditis elegans* has made it a successful tool for genetic studies, as it presents over 60% of homologous genes to human disease genes [5], and more recently, it has been considered in research on the complex pathways involved in NDDs [6]. In particular, polyQ expansions expressed in *C. elegans* provide a model system to study protein misfolding-induced toxicity, the pathways involved, and the treatment and/or prevention strategies that may be adopted against these debilitating diseases [7].

**Figure 1:**
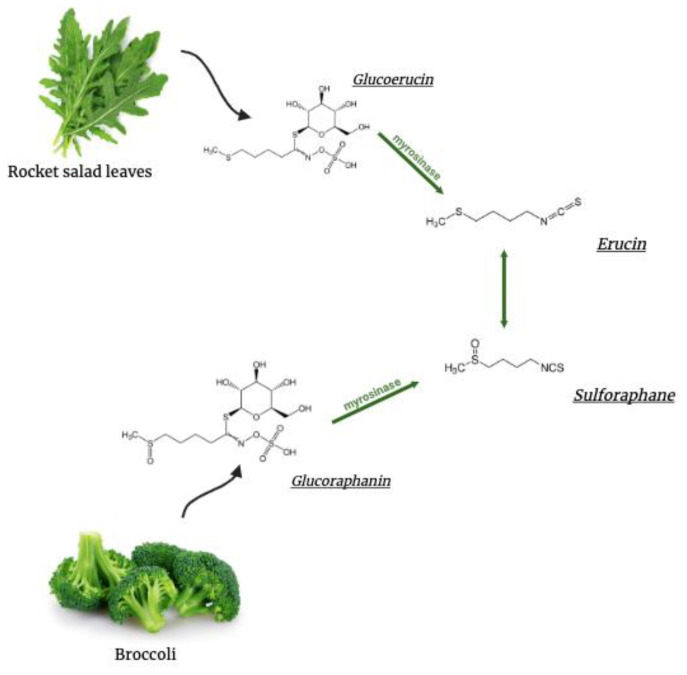
Pharmacokinetics and bioavailability of ERN. The isothiocyanate ERN is a reduced analog of the isothiocyanate SFN. It is released both from the enzymatic hydrolysis of glucoerucin, a GL found in rocket salad leaves (*Eruca sativa* Mill., *Diplotaxis tenuifolia* L.) and from the in vivo reduction of SFN, derived from the conversion of glucoraphanin, the GL typical of broccoli (*Brassica oleracea* L. ssp italica). Myrosinase is the enzyme responsible for the hydrolysis of GLs.

Natural compounds capable of preventing the onset or mitigating the phenotype of protein aggregation-related NDDs are currently of great interest, and glucosinolates (GLs) and their related hydrolytic products, isothiocyanates (ITCs), have been reported to exert a protective effect against the development of NDDs [8]. In particular, the beneficial role of ITCs could be ascribed to their antioxidant properties, considering that ROS production and oxidative stress have been suggested to play a crucial role in NDD progression when the so-called “unfolded protein response” is insufficient to restore protein homeostasis and the redox status of cells [9]. According to current knowledge, the main neuroprotective potential of GLs is ascribed to their ability to enhance total glutathione levels and related enzymes through the activation of the nuclear factor erythroid 2-related factor 2 (Nrf2) [10]. In this regard, our study focused on 4-(methylthio)butyl isothiocyanate, erucin (ERN), an ITC released through the enzymatic hydrolysis of its precursor molecule (glucoerucin), naturally present in rocket salad leaves (*Eruca sativa* Mill., *Diplotaxis tenuifolia* L.) [11]. Recent bioavailability studies in mice and human subjects indicate that ERN is also released through the in vivo reduction of 4-(methylsulfinyl)butyl isothiocyanate, sulforaphane (SFN), which represents its oxidized form, typical of broccoli (*Brassica oleracea* L. ssp. italica) (Figure 1) [12,13]. Additionally, another study in human subjects shows that human colonic flora contributes to the conversion of SFN into ERN [14].

The choice of this natural compound was justified by the presence of in vitro and in vivo studies that had already demonstrated its anti-inflammatory, antioxidant, and neuroprotective properties via the c-Jun N-terminal kinases, the extracellular signal-regulated kinase 1/2, and the mitogen-activated protein kinases p38 signaling transduction pathways, which are able to enhance nuclear Nrf2 levels [11,15,16]. In lipopolysaccharide (LPS)-challenged umbilical vein endothelial cells, ERN reduced the increase in reactive oxygen species and tumor necrosis factor α and decreased cyclooxygenase 2, even increasing nuclear factor kappa B (NF-kβ) levels [17]. Moreover, ERN, as a hydrogen sulfide (H_2_S) donor, has exerted neuroprotective effects in an LPS-induced microglia inflammation model and elicited a decrease in nitric oxide levels in Alzheimer’s disease LPS-stimulated cells [18]. Finally, because of their molecular interchangeability, ERN has shown in vitro and in vivo effects similar to those of SFN in SH-SY5Y cells simultaneously treated with 6-hydroxydopamine (6-OHDA) and in a 6-OHDA Parkinson’s disease (PD) mouse model, respectively [19].

Therefore, the aim of our study was to investigate ERN’s modulating action against protein aggregation-related NDDs in different tissues in *C. elegans* and the signal transduction pathways involved, given that these signaling pathways are very well conserved between mammals and nematodes. In this regard, *C. elegans* is a valuable organism model for studying the bioactive effects of food-derived components in NDDs [20,21]. These effects often influence the insulin/IGF-1 and AMP-activated protein kinase (AMPK) signaling pathways, typically resulting in the upregulation of *daf-16*, which is the ortholog of the FOXO gene family [20,22,23]. Additionally, these nematodes can be used as preliminary models to investigate how ERN may function in living organisms before planning experiments in in vivo rodent models [20].

Our results demonstrate that ERN is able to prevent muscular and neuronal polyQ-induced toxicity in *C. elegans* via *aak-2*/AMPK signaling and, downstream in the pathway, through the *daf-16*/FOXO transcription factor. We finally report that ERN prevents pharyngeal aggregate accumulation and slightly restores motility impairment in a PD *C. elegans* model.

## 2. Results and Discussion

### 2.1. Erucin Reduces Polyglutamine-Induced Toxicity

**Figure 2:**
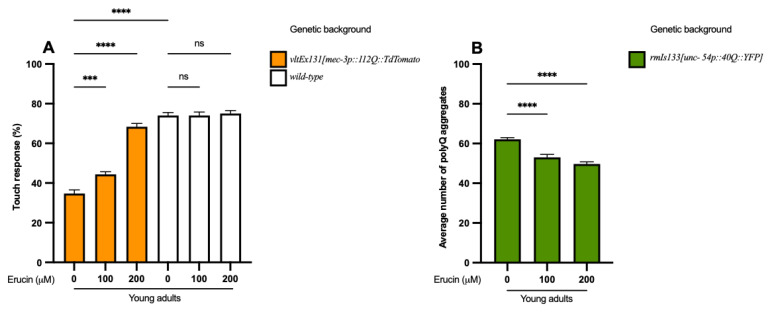
Erucin restores neuronal function and reduces polyglutamine-induced muscular toxicity. (**A**) The expression of 112Q:TdTom in mechanosensory neurons induces an impairment of neuronal function compared to animals that do not have the transgene. Treatment in N2 wild-type worms does not affect the rate of response to touch for each concentration of ERN tested compared to untreated animals. Treatment with 100 μM and 200 μM ERN rescues neuronal function in 112Q: TdTom young adult animals compared to untreated nematodes. (**B**) Erucin treatment reduces polyQ-induced muscular aggregation in 40Q young adult animals significantly. *** *p* < 0.001, **** *p* < 0.0001 and ns, not significant.

**Figure 3:**
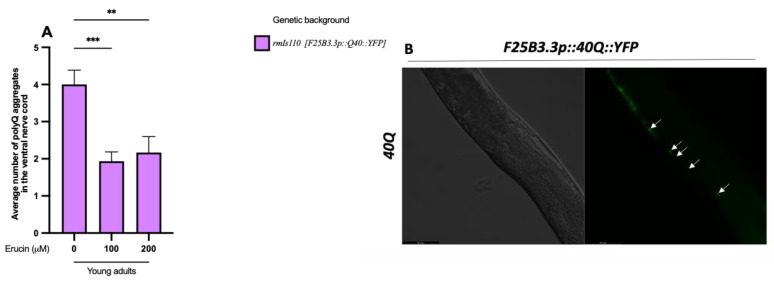
Erucin reduces polyglutamine-induced neuronal toxicity. (**A**) Erucin treatment reduces polyQ-induced aggregate formation in the ventral nerve cord in 40Q young adult animals. (**B**) Representative images from fluorescence microscopy showing five neuronal aggregates of polyQs (white arrows) in the ventral nerve cord of *F25B3.3p::40Q::YFP* young adult animals at 20× magnification. ** *p* < 0.01, *** *p* < 0.001.

We initially evaluated the ability of ERN to suppress polyQ-induced toxicity in vivo in *C. elegans*. Then, we assessed the mechanism behind the reduction in polyQ toxicity mediated by ERN. To achieve this, we utilized a *C. elegans* model of neuronal toxicity that carries a transgene encoding a 112Q fusion with the dimeric fluorescent protein TdTomato (TdTom) in mechanosensory neurons, which causes impairment in their function. The advantage of this model relies on the possibility of testing neuronal functionality by lightly touching the posterior portion of the nematode with an eyelash fused on the top of a pipette tip. These nematodes usually respond only 35% of the time, while wild-type worms respond 7 out of 10 times (*p* < 0.0001) (Figure 2A). We cultured worms with 100 µM and 200 µM ERN separately, both for the N2 wild-type and for the TdTom strain, and we found that ERN was able to reduce polyQ-induced toxicity rescuing neuronal functionality compared to untreated nematodes (*p* < 0.001 for 100 µM ERN and *p* < 0.0001 for 200 µM ERN) (Figure 2A). Moreover, we demonstrated that the same treatment in N2 wild-type worms did not affect the normally observed rate of response to the touch for each concentration of ERN tested (Figure 2A). Therefore, we tested whether this compound was able to interfere with polyQ-induced aggregate formation to determine if the neuroprotection given by ERN was connected to mechanisms for clearing polyQ aggregates. To test this hypothesis, we used worms that expressed 40 CAG repeats in frame with the yellow fluorescence protein (40Q::YFP) in muscle cells that accumulate polyQ aggregates in an age-dependent manner. The treatment with both 100 µM and 200 µM ERN reduced the number of polyQ aggregates significantly compared to untreated young adult animals (*p* < 0.0001 for 100 µM and 200 µM ERN) (Figure 2B). Once we verified that ERN was able to interact with polyQ accumulation in muscle cells, we proposed to test if the compound showed the same ability in the neuronal tissue. In this regard, we used worms expressing 40 CAG repeats in frame with the yellow fluorescence protein (40::YFP) in neuronal cells, which induced an accumulation of polyQ aggregates in the ventral nerve cord, demonstrating that ERN reduced even the neuronal aggregate formation in 40::YFP worms (*p* < 0.001 for 100 µM ERN and *p* < 0.01 for 200 µM ERN) (Figure 3A). Figure 3B represents the aggregates accumulated in the ventral nerve cord of an explicative young adult 40::YFP nematode.

These results confirm recent in vitro and in vivo studies supporting the idea that isothiocyanates (ITCs) can enhance the clearance of polyQ aggregates, particularly in Huntington’s disease (HD). Specifically, SFN has been shown to facilitate the degradation of mutant huntingtin protein fragments with expanded polyglutamine primarily through the ubiquitin–proteasome system pathway in mice and Huntington’s disease cells [24]. Additionally, SFN exhibited similar effects in normal human fibroblasts expressing mutated huntingtin by activating AMPK, which subsequently promoted autophagy [25]. Furthermore, recent studies indicate that various small-molecule Nrf2 activators can alleviate polyglutamine toxicity in conditions such as SBMA [26]. This suggests that activating the Nrf2 pathway may be a potential pharmacological intervention for SBMA and possibly other polyglutamine diseases. Given this context, all ITCs, including ERN, can activate the Nrf2 pathway, which may allow them to share this mechanism to combat polyglutamine toxicity effectively.

Our findings indicate that ERN reduces polyQ-induced toxicity at both muscular and neuronal levels, providing further insights into the efficacy of this natural compound.

### 2.2. Reduction in polyQ Aggregation Induced by Erucin Requires AMPK Catalytic Function and daf-16/FOXO Transcription Factor

These results gave us a justifiable basis to investigate the pathways involved in the mechanism of action of ERN. Considering that SFN anti-oxidative action and autophagy are mediated by the activation of the AMPK signaling pathway in vitro [27] and in vivo [28,29], we proposed to test whether a reduction in the number of aggregates in 40Q::YFP worms treated with ERN required AMPK function. Moreover, it has been demonstrated that polyQ toxicity can be avoided by AMPK activation in *C. elegans* [30] as it prevents neurons from the dysfunction induced by human exon-1 huntingtin expression in a *daf-16*/forkhead box O-dependent manner (FOXO) [31].

**Figure 4:**
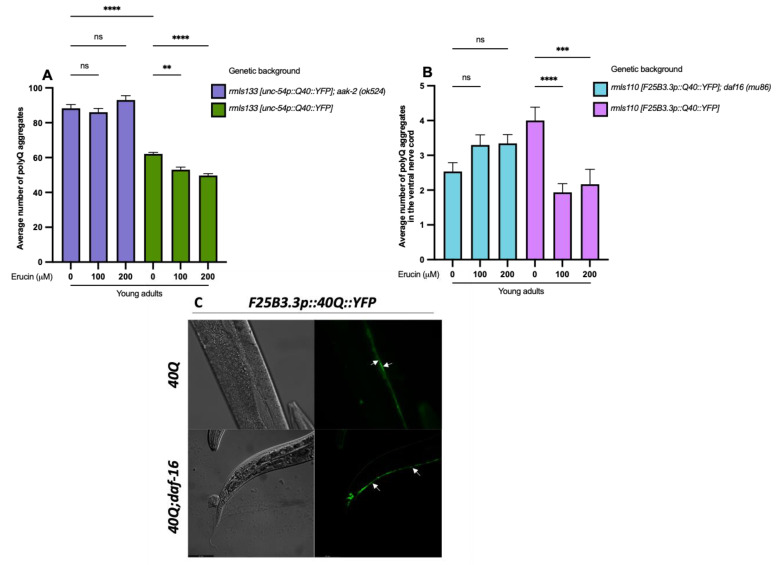
Reduction in polyQ aggregation induced by erucin requires AMPK catalytic function and *daf-16*/FOXO transcription factor. (**A**) The treatment with 100 µM and 200 µM ERN of 40Q::YFP worms defective of the catalytic subunit of AMPK (*aak-2*/AMPKα2) does not produce any significant difference in polyQ-induced muscular aggregate formation in comparison to untreated worms. In parallel, ERN treatment reduces polyQ-induced muscular aggregation in 40Q young adult animals of the control strain significantly. (**B**) The treatment with 100 µM and 200 µM ERN of 40Q::YFP worms defective of the *daf-16* gene does not produce any significant difference in polyQ-induced neuronal aggregate formation in comparison to untreated worms. In parallel, ERN treatment significantly reduces polyQ-induced muscular aggregation in 40Q young adult animals of the control strain. (**C**) Representative images from fluorescence microscopy showing neuronal aggregates of polyQs (white arrows) in the ventral nerve cord of *F25B3.3p::40Q::YFP* and *F25B3.3p::40Q::YFP; daf-16(mu86)* young adult animals at 20× magnification. In each fluorescent image, two neuronal aggregates are counted and indicated by white arrows, showing no difference between the control strain (*F25B3.3p::40Q::YFP*) and the mutated one (*F25B3.3p::40Q::YFP; daf-16(mu86)*). ** *p* < 0.01, *** *p* < 0.001, **** *p* < 0.0001 and ns, not significant.

Therefore, we treated worms defective of the only catalytic subunit of AMPK (AMPKα) that affected the lifespan and health in *C. elegans* (*aak-2*/AMPKα2) [32]. Analysis of 40Q; *aak-2(ok524)* young adults showed no difference in the number of polyQ aggregates in nematodes treated with 100 µM and 200 µM ERN in comparison to untreated ones. Considering that ERN treatment reduced polyQ muscular aggregation in 40Q young adult animals of the control strain without the mutation significantly (*p* < 0.01 for 100 µM ERN and *p* < 0.0001 for 200 µM ERN) (Figure 4A), we demonstrated the involvement of AMPK in the mechanism of action of ERN.

Moreover, there is evidence that *daf-16/*FOXO exerts a strong protective action in *C. elegans* models of HD [33] and that FOXO factors are involved in the AMPK mechanism of action [31,32]. Therefore, considering that *daf-16*/FOXO is involved in SFN promotion of longevity and lifespan in *C. elegans* [34], we aimed to investigate whether the neuroprotection exerted by ERN through AMPK might be dependent on *daf-16*/FOXO.

To verify this hypothesis, we treated 40Q::YFP nematodes defective of the *daf-16* gene with 100 µM and 200 µM ERN. We found that the treatment did not produce any significant variation in aggregate accumulation in the ventral nerve cord of the worms. In parallel, the treatment of the relative control strain without mutation induced a significant reduction in the number of aggregates for both concentrations of ERN tested (*p* < 0.0001 for 100 µM ERN and *p* < 0.001 for 200 µM ERN) (Figure 4B). From these results, it was evident that the aggregation in the *rmls110; daf-16* strain was reduced in comparison to the *rmls110* strain: we hypothesize that this unexpected trend appeared because of a change in the genetic backgrounds during the crossing process or because worms had different growing times and the *rmls110; daf-16* young animals were slightly younger than those of the *rmls110* strain, consequently with fewer aggregates. However, beyond the differences between the two strains, the important result was that the rescuing effect was lost in the strain carrying the mutation, which demonstrated that the *daf-16*/FOXO factor is involved in ERN neuroprotective action.

Taken together, these results evidence that ERN prevents polyQ-induced aggregate formation both in muscular and neuronal tissues. This occurs through the activation of the AMPK catalytic subunit, which in turn induces the expression of the *daf-16*/FOXO transcription factor. This finding supports previous in vitro [27] and in vivo [28,29,34] evidence regarding the involvement of AMPK and *daf-16*/FOXO in the mechanism of action of SFN, the oxidized ERN molecule. Moreover, these results do not confirm that *aak-2* and *daf-16* operate in the same signaling pathway. Double mutants *40Q; aak-2; daf-16* are not viable in the strain expressing 40 CAG repeats in muscle cells. Nevertheless, it has already been demonstrated in a 128Q strain expressing impairment in the touch response that the reaction to the stimulus in *128Q; daf-16* nematodes is comparable to that of *128Q; aak-2* worms and that double mutants *128Q; aak-2; daf-16* do not experience an additive effect, demonstrating that AMPK and *daf-16*/FOXO operate in the same signaling pathway [31]. Therefore, it is reasonable to assume that these factors are involved in the same axis even in the 40Q::YFP nematodes.

### 2.3. α-Synuclein Aggregation Pattern Is Rescued by Erucin

**Figure 5:**
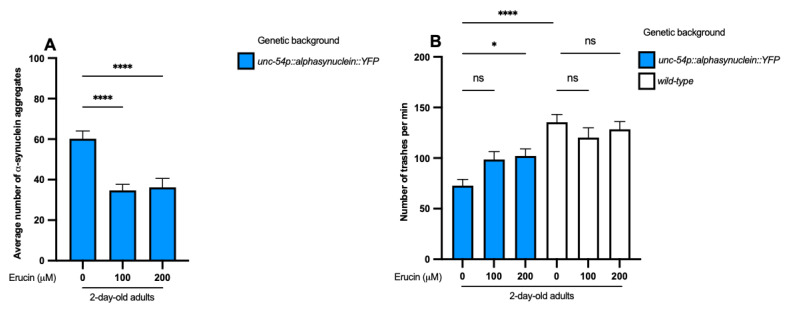
Treatment with erucin reduces α-synuclein aggregation and slightly restores motor capacity. (**A**) Treated animals with 100 µM and 200 µM ERN show a reduction in α-syn aggregates compared to untreated α-syn::YFP 2-day-old adult animals. (**B**) α-syn aggregates induce an impairment of motility compared with wild-type animals. Treated wild-type worms with both concentrations of ERN tested did not show motility impairment. Treated α-syn::YFP nematodes with 100 µM and 200 µM ERN rescue motility capacity of 2-day-old adult animals significantly after treatment only with the higher dose of ERN tested. * *p* < 0.05, **** *p* < 0.0001 and ns, not significant.

To broaden the range of NDDs included in our study, we aimed to verify if ERN could interfere with the toxicity induced by α-synuclein (α-syn). To do so, we used the well-known strain that expresses α-syn in muscle cells [35]. As the characteristic of the strain is to accumulate aggregates in 2-day-old adults, we prolonged the treatment with 100 µM and 200 µM ERN from the L1 to the second day of adulthood. We performed the count of the aggregates in the area included between the two bulbs of the pharynx through a fluorescence microscope, as described elsewhere [36]. The analysis showed a significant reduction in muscular α-syn aggregates with both concentrations of ERN tested (*p* < 0.0001 for 100 µM and 200 µM ERN) (Figure 5A). Moreover, we wanted to test whether ERN treatment improved the behavior of α-syn::YFP worms through the trashing assay. In parallel, we performed the same test on N2 wild-type animals to demonstrate that ERN did not affect the natural movement of worms (Figure 5B). When compared to wild-type nematodes, untreated α-syn::YFP showed a significant reduction in their motility capacity (*p* < 0.0001). In contrast, only a slightly significant recovery of movement was guaranteed by 200 µM ERN on α-syn::YFP nematodes compared to untreated worms (*p* < 0.05 for 200 µM ERN) (Figure 5B). These results suggest that a higher concentration of the compound could be more effective in rescuing the impaired thrashing due to α-syn aggregates accumulation. Simultaneously, they demonstrate that ERN reduces the aggregation of the protein linked genetically and neuropathologically to PD. The inclusion of the PD model in our study was justified by several studies that had already demonstrated that AMPK, as one of the key regulators of autophagy, a conservative intracellular degradation process, lowers protein aggregation (i.e., α-syn aggregates) protecting cells from impairment and degeneration [37,38,39]; moreover, *daf-16* signaling had been reported to be involved in the reduction in α-syn-induced aggregate formation [40]. This evidence suggests a signaling pathway similar to that of polyQ-induced toxicity clearance in the *C. elegans* model of PD, supporting further studies to test this hypothesis.

## 3. Materials and Methods

### 3.1. Chemicals

ERN was purchased from LKT Laboratories (LKT Laboratories, St. Paul, MN, USA). The ERN stock solution was prepared in dimethyl sulfoxide (DMSO) at 10 mM. The stock solutions were diluted in M9 1X Buffer as a solvent to obtain the requested concentrations of ERN in a maximum of 0.1% DMSO.

### 3.2. C. elegans Strains

The strains N2 standard wild-type, AM141: *rmIs133[unc-54p::40Q::YFP]*, and AM101: *rmIs110[F25B3.3p::Q40::YFP]* were obtained from the Caenorhabditis Genetics Center (CGC Elegans) (University of Minnesota, MN, USA). The strains RVM455: *rmIs133[unc-54p::Q40::YFP]*; *aak-2(ok524)*, RVM131: *vltEx131[mec-3p::112Q::TdTomato; myo-2p::GFP]*, RVM584 *rmIs110[F25B3.3p::Q40::YFP]*; and *daf-16(mu86)*, NL5901: *pkIs2386 [unc-54p::alphasynuclein::YFP + unc-119(+)]* were obtained from the Laboratory of Molecular, Cellular and Genomic Biomedicine, Instituto De Investigación Sanitaria La Fe, Valencia, Spain; Joint Unit for Rare Diseases IIS La Fe-CIPF, 46012 Valencia, Spain.

### 3.3. Culture and Sampling of C. elegans

All strains were grown in NGM (0.3% NaCl, 1.7% agar, 0.3% peptone, 5 mg/mL cholesterol, 1 M KPO_4_, 1 M MgSO_4_) agar plates and fed with the bacterium *Escherichia coli* OP50 strain (CGC Elegans, but cultivated for a while in the Laboratory of Molecular, Cellular and Genomic Biomedicine, Instituto De Investigación Sanitaria La Fe, Valencia, Spain; Joint Unit for Rare Diseases IIS La Fe-CIPF, 46012 Valencia, Spain), distributed in a thin layer over the agar. All worm strains were maintained at 20 °C degrees, according to the standard protocol as described elsewhere [41].

### 3.4. Treatment of C. elegans with Erucin

In order to perform the treatment of the strains, two ERN concentrations (100 µM and 200 µM) were selected, which were considered not toxic by lifespan assays performed previously.

NGM agar plates (60 mm) were seeded with *E. coli* strain OP50 as a food source and with 300 µL of 100 µM and 200 µM ERN. ERN solutions were prepared by diluting ERN stock (10 mM) in M9.

To treat the worms in solid culture, ten 1-day-adult worms 4 days after egg hatching were transferred to NGM agar plates, with or without ERN at the two selected concentrations, and allowed to lay eggs for 4 h. Then, the nematodes were removed. Five hours later—with small variations depending on the worm strain—larvae at stage 1 (L1) hatched. After the development of young adults, approximately 3 days later, we scored the number of polyQ aggregates on the 40Q::YFP worms and assayed the touch response on 112Q::TdTom nematodes. Moreover, as worms expressing the α-syn construct tended to form aggregates, we scored the number of α-syn aggregates and evaluated motility in 2-day-old adult animals, approximately 5 days after the hatching of eggs. Worms were maintained at 20 °C until they reached young adulthood or the 2-day-old adult stage.

### 3.5. In Vivo Scoring of polyQ Aggregates in Muscle Cells

The production of muscular polyQ aggregates induced by the 40Q::YFP transgene is age-dependent and can be evaluated through a dissecting microscope equipped with fluorescence. At least ten young adults were analyzed for each genotype and each independent experiment for each condition (untreated/treated worms) using an M165FC Leica dissecting microscope (Leica Microsystems GmbH, Wetzlar, Germany). The analysis was repeated in three independent experiments for a total number of at least 30 worms.

### 3.6. In Vivo Scoring of polyQ Aggregates in the Ventral Nerve Cord

The production of neuronal polyQ aggregates induced by the 40Q::YFP transgene is age-dependent but is not appreciable through a dissecting microscope. For this reason, the analysis was performed with a THUNDER-equipped Leica DM6 B Upright Microscope with a Leica DMC5400 Camera and LAS X Office software, version 1.4.7 28921 (Leica Microsystems GmbH, Wetzlar, Germany). At least ten young adults were analyzed for each genotype and each independent experiment for each condition (untreated/treated worms). The analysis was repeated in three independent experiments for a total number of at least 30 worms.

### 3.7. Evaluation of the Touch Response

The accumulation of polyQ aggregates in mechanosensory neurons produces a touch response dysfunction in 112Q::TdTomato worms [42]. Positive responses to a light touch in these neurons induce the worms to move forward. The touch response of at least 30 young adult animals was recorded by gently touching the posterior part of the nematodes through an eyelash fused on the top of a pipette tip. Each animal was touched ten times, and we here represent the percentage of times the animal responded to the touch, as described elsewhere [43]. Each test was repeated in three independent experiments in the 112Q::TdTomato strain and in the N2 wild-type for each condition. The touch response phenotype was scored using a Leica MS5 dissecting microscope (Leica Microsystems GmbH, Wetzlar, Germany).

### 3.8. In Vivo Scoring of α-Synuclein Aggregates

Differently from polyQ worms that express aggregates in muscular and neuronal cells already in young adulthood, animals carrying the α-synuclein::YFP transgene accumulate aggregation in old worms (2-day-old adult animals or older). Therefore, we prolonged the treatment with 100 µM and 200 µM ERN until the second day of adulthood of worms and performed the analysis, selecting the area between the two pharyngeal bulbs since the counting of aggregates in the whole muscle tissue is not feasible, as described elsewhere [36]. The assay was performed with a THUNDER-equipped Leica DM6 B Upright Microscope with a Leica DMC5400 Camera at 63× magnification. We analyzed 30 untreated/treated 2-day-old adult animals in total per condition, and we performed each experiment three times.

### 3.9. Motility Assay

To evaluate the behavioral effect of ERN, we assessed the motility capacity of worms expressing the α-synuclein::YFP gene, counting the number of thrashes per treated animal for 30 s and extrapolating the average data to represent thrashes per minute. Before scoring, each animal was immersed in M9 solution for 30 s to let it acclimatize to the liquid medium, as described elsewhere [36]. We analyzed at least 10 untreated/treated 2-day-old adult animals per condition. We performed the same analysis to confirm our data even on the N2 wild-type strain. We performed each experiment three times to obtain reproducible values for a total number of at least 30 animals per condition.

### 3.10. Fluorescent Microscopy Imaging

Images were collected using a THUNDER-equipped Leica DM6 B Upright Microscope with a Leica DMC5400 Camera and LAS X Software. Live animals were selected and positioned above 2% agarose pads inside a drop of 0.25 M sodium azide to anesthetize them.

### 3.11. Statistical Analysis

One-way analysis of variance (one-way ANOVA) was applied to the data of every trial to study the effect of ERN treatment at the concentrations selected. In particular, Dunnett’s method for multiple comparisons was applied to compare the mean of each treated group and the mean of the control group for each strain. GraphPad software (version 10.1.1) was used to perform the statistical analyses. The results are presented with the relative *p*-value to indicate the significance of the data.

## 4. Conclusions

Our study shows that ERN reduces the neurotoxic effects of expanded polyQs and α-syn in vivo. Moreover, our results demonstrate that ERN activity is AMPK-dependent and that, downstream in the pathway, it implies *daf-16*/FOXO signaling transduction. These findings provide a basis for testing whether other signaling pathways related to AMPK are involved in the neuroprotective action of ERN. Given that AMPK and the signaling pathways connected to it are strongly conserved in mammals and *C. elegans*, ERN could be a potential neuroprotective compound even in humans.

Regarding the neuroprotective effects of ERN in *C. elegans*, limitations of this model organism should be considered, such as its simple nervous system without a myelin membrane and the lack of some mammalian anatomical features, including a circulatory system and blood–brain barrier [21,44]. However, several bioavailability studies in mice demonstrated the distribution of ITC metabolites in several tissues, including the brain, supporting the potential ability of ERN to cross the blood–brain barrier and reach the brain [45]. Remarkably, ERN data from the in silico evaluation by SwissADME, a predictive model for pharmacokinetics and drug-likeness of small molecules, show that this ITC has a high probability of crossing both the gastrointestinal and blood–brain barriers (Supplementary Table S1) [46]. Therefore, further pre-clinical studies in mice models of protein aggregation are justified and could provide insights to validate this hypothesis.

## Data Availability

The data presented in this study are available on request from the corresponding author.

## Supplementary Materials

The following supporting information can be downloaded at https://www.mdpi.com/article/10.3390/ijms252212220/s1.

## References

[1] Paulson H. (2018). Repeat expansion diseases. Handb. Clin. Neurol..

[2] Lieberman A.P., Shakkottai V.G., Albin R.L. (2019). Polyglutamine Repeats in Neurodegenerative Diseases. Annu. Rev. Pathol..

[3] Tenchov R., Sasso J.M., Zhou Q.A. (2024). Polyglutamine (PolyQ) Diseases: Navigating the Landscape of Neurodegeneration. ACS Chem. Neurosci..

[4] Kopito R.R. (2000). Aggresomes, inclusion bodies and protein aggregation. Trends Cell Biol..

[5] Caldero-Escudero E., Romero-Sanz S., De la Fuente S. (2024). Using *C. elegans* as a model for neurodegenerative diseases: Methodology and evaluation. Methods Cell Biol..

[6] Caldwell K.A., Willicott C.W., Caldwell G.A. (2020). Modeling neurodegeneration in *Caenorhabditis elegans*. Dis. Models Mech..

[7] Satyal S.H., Schmidt E., Kitagawa K., Sondheimer N., Lindquist S., Kramer J.M., Morimoto R.I. (2000). Polyglutamine aggregates alter protein folding homeostasis in *Caenorhabditis elegans*. Proc. Natl. Acad. Sci. USA.

[8] Jaafaru M.S., Karim N.A.A., Enas M.E., Rollin P., Mazzon E., Razis A.F.A. (2018). Protective Effect of Glucosinolates Hydrolytic Products in Neurodegenerative Diseases (NDDs). Nutrients.

[9] Calabrese V., Guagliano E., Sapienza M., Panebianco M., Calafato S., Puleo E., Pennisi G., Mancuso C., Butterfield D.A., Stella A.G. (2007). Redox regulation of cellular stress response in aging and neurodegenerative disorders: Role of vitagenes. Neurochem. Res..

[10] Giacoppo S., Galuppo M., Montaut S., Iori R., Rollin P., Bramanti P., Mazzon E. (2015). An overview on neuroprotective effects of isothiocyanates for the treatment of neurodegenerative diseases. Fitoterapia.

[11] Melchini A., Traka M.H. (2010). Biological profile of erucin: A new promising anticancer agent from cruciferous vegetables. Toxins.

[12] Bricker G.V., Riedl K.M., Ralston R.A., Tober K.L., Oberyszyn T.M., Schwartz S.J. (2014). Isothiocyanate metabolism, distribution, and interconversion in mice following consumption of thermally processed broccoli sprouts or purified sulforaphane. Mol. Nutr. Food Res..

[13] Clarke J.D., Hsu A., Riedl K., Bella D., Schwartz S.J., Stevens J.F., Ho E. (2011). Bioavailability and inter-conversion of sulforaphane and erucin in human subjects consuming broccoli sprouts or broccoli supplement in a cross-over study design. Pharmacol. Res..

[14] Saha S., Hollands W., Teucher B., Needs P.W., Narbad A., Ortori C.A., Barrett D.A., Rossiter J.T., Mithen R.F., Kroon P.A. (2012). Isothiocyanate concentrations and interconversion of sulforaphane to erucin in human subjects after consumption of commercial frozen broccoli compared to fresh broccoli. Mol. Nutr. Food Res..

[15] Tarozzi A., Morroni F., Bolondi C., Sita G., Hrelia P., Djemil A., Cantelli-Forti G. (2012). Neuroprotective effects of erucin against 6-hydroxydopamine-induced oxidative damage in a dopaminergic-like neuroblastoma cell line. Int. J. Mol. Sci..

[16] Wagner A.E., Sturm C., Piegholdt S., Wolf I.M., Esatbeyoglu T., De Nicola G.R., Iori R., Rimbach G. (2015). Myrosinase-treated glucoerucin is a potent inducer of the Nrf2 target gene heme oxygenase 1—Studies in cultured HT-29 cells and mice. J. Nutr. Biochem..

[17] Ciccone V., Piragine E., Gorica E., Citi V., Testai L., Pagnotta E., Matteo R., Pecchioni N., Montanaro R., Mannelli L.D.C. (2022). Anti-Inflammatory Effect of the Natural H2S-Donor Erucin in Vascular Endothelium. Int. J. Mol. Sci..

[18] Sestito S., Pruccoli L., Runfola M., Citi V., Martelli A., Saccomanni G., Calderone V., Tarozzi A., Rapposelli S. (2019). Design and synthesis of H2S-donor hybrids: A new treatment for Alzheimer’s disease?. Eur. J. Med. Chem..

[19] Morroni F., Sita G., Djemil A., D’Amico M., Pruccoli L., Cantelli-Forti G., Hrelia P., Tarozzi A. (2018). Comparison of Adaptive Neuroprotective Mechanisms of Sulforaphane and its Interconversion Product Erucin in in Vitro and in Vivo Models of Parkinson’s Disease. J. Agric. Food Chem..

[20] Mudd N., Liceaga A.M. (2022). *Caenorhabditis elegans* as an in vivo model for food bioactives: A review. Curr. Res. Food Sci..

[21] Roussos A., Kitopoulou K., Borbolis F., Palikaras K. (2023). *Caenorhabditis elegans* as a Model System to Study Human Neurodegenerative Disorders. Biomolecules.

[22] Burkewitz K., Weir H.J.M., Mair W.B. (2016). AMPK as a Pro-longevity Target. Exp. Suppl..

[23] Rashid S., Wong C., Roy R. (2021). Developmental plasticity and the response to nutrient stress in *Caenorhabditis elegans*. Dev. Biol..

[24] Liu Y., Hettinger C.L., Zhang D., Rezvani K., Wang X., Wang H. (2014). Sulforaphane enhances proteasomal and autophagic activities in mice and is a potential therapeutic reagent for Huntington’s disease. J. Neurochem..

[25] Brokowska J., Hać A., Węgrzyn G., Herman-Antosiewicz A. (2016). L12 Sulforaphane reduces the level of exogenous mutated huntingtin protein in normal human fibroblasts. J. Neurol. Neurosurg. Psychiatry.

[26] Bott L.C., Badders N.M., Chen K.-L., Harmison G.G., Bautista E., Shih C.C.-Y., Katsuno M., Sobue G., Taylor J.P., Dantuma N.P. (2016). A small-molecule Nrf1 and Nrf2 activator mitigates polyglutamine toxicity in spinal and bulbar muscular atrophy. Hum. Mol. Genet..

[27] Taheri M., Roudbari N.H., Amidi F., Parivar K. (2022). Investigating the effect of Sulforaphane on AMPK/AKT/NRF2 pathway in human granulosa-lutein cells under H_2_O_2_-induced oxidative stress. Eur. J. Obstet. Gynecol. Reprod. Biol..

[28] Masuda M., Yoshida-Shimizu R., Mori Y., Ohnishi K., Adachi Y., Sakai M., Kabutoya S., Ohminami H., Yamanaka-Okumura H., Yamamoto H. (2022). Sulforaphane induces lipophagy through the activation of AMPK-mTOR-ULK1 pathway signaling in adipocytes. J. Nutr. Biochem..

[29] Yang G., Lee H.E., Lee J.Y. (2016). A pharmacological inhibitor of NLRP3 inflammasome prevents non-alcoholic fatty liver disease in a mouse model induced by high fat diet. Sci. Rep..

[30] Gómez-Escribano A., Bono-Yagüe J., García-Gimeno M., Sequedo M.D., Hervás D., Fornés-Ferrer V., Torres-Sánchez S., Millán J., Sanz P., Vázquez-Manrique R. (2020). Synergistic activation of AMPK prevents from polyglutamine-induced toxicity in *Caenorhabditis elegans*. Pharmacol. Res..

[31] Vázquez-Manrique R.P., Farina F., Cambon K., Sequedo M.D., Parker A.J., Millán J.M., Weiss A., Déglon N., Neri C. (2016). AMPK activation protects from neuronal dysfunction and vulnerability across nematode, cellular and mouse models of Huntington’s disease. Hum. Mol. Genet..

[32] Apfeld J., O’Connor G., McDonagh T., DiStefano P.S., Curtis R. (2004). The AMP-activated protein kinase AAK-2 links energy levels and insulin-like signals to lifespan in *C. elegans*. Genes Dev..

[33] Parker J.A., Vazquez-Manrique R.P., Tourette C., Farina F., Offner N., Mukhopadhyay A., Orfila A.-M., Darbois A., Menet S., Tissenbaum H.A. (2012). Integration of β-catenin, sirtuin, and FOXO signaling protects from mutant huntingtin toxicity. J. Neurosci..

[34] Qi Z., Ji H., Le M., Li H., Wieland A., Bauer S., Liu L., Wimk M., Herr I. (2021). Sulforaphane promotes *C. elegans* longevity and healthspan via DAF-16/DAF-2 insulin/IGF-1 signaling. Aging.

[35] van Ham T.J., Thijssen K.L., Breitling R., Hofstra R.M.W., Plasterk R.H.A., Nollen E.A.A. (2008). *C. elegans* model identifies genetic modifiers of alpha-synuclein inclusion formation during aging. PLoS Genet..

[36] Gómez-Escribano A.P., Mora-Martínez C., Roca M., Walker D.S., Panadero J., Sequedo M.D., Saini R., Knölker H., Blanca J., Burguera J. (2023). Changes in lipid metabolism driven by steroid signalling modulate proteostasis in *C. elegans*. EMBO Rep..

[37] Curry D.W., Stutz B., Andrews Z.B., Elsworth J.D. (2018). Targeting AMPK Signaling as a Neuroprotective Strategy in Parkinson’s Disease. J. Park. Dis..

[38] Hang L., Wang Z., Foo A.S., Goh G.W., Choong H.C., Thundyil J., Xu S., Lam K.-P., Lim K.-L. (2021). Conditional disruption of AMP kinase in dopaminergic neurons promotes Parkinson’s disease-associated phenotypes in vivo. Neurobiol. Dis..

[39] Parekh P., Sharma N., Sharma M., Gadepalli A., Sayyed A.A., Chatterjee S., Kate A., Khairnar A. (2022). AMPK-dependent autophagy activation and alpha-Synuclein clearance: A putative mechanism behind alpha-mangostin’s neuroprotection in a rotenone-induced mouse model of Parkinson’s disease. Metab. Brain Dis..

[40] Chen Y., Xu R., Liu Q., Zeng Y., Chen W., Liu Y., Cao Y., Liu G., Chen Y. (2024). Rosmarinic acid ameliorated oxidative stress, neuronal injuries, and mitochondrial dysfunctions mediated by polyglutamine and α-synuclein in *Caenorhabditis elegans* models. Mol. Neurobiol..

[41] Stiernagle T. (2006). Maintenance of *C. elegans*. WormBook.

[42] Sanchis A., García-Gimeno M.A., Cañada-Martínez A.J., Sequedo M.D., Millán J.M., Sanz P., Vázquez-Manrique R.P. (2019). Metformin treatment reduces motor and neuropsychiatric phenotypes in the zQ175 mouse model of Huntington disease. Exp. Mol. Med..

[43] Parker J.A., Connolly J.B., Wellington C., Hayden M., Dausset J., Neri C. (2001). Expanded polyglutamines in *Caenorhabditis elegans* cause axonal abnormalities and severe dysfunction of PLM mechanosensory neurons without cell death. Proc. Natl. Acad. Sci. USA.

[44] Tissenbaum H.A. (2015). Using *C. elegans* for aging research. Invertebr. Reprod. Dev..

[45] Clarke J.D., Hsu A., Williams D.E., Dashwood R.H., Stevens J.F., Yamamoto M., Ho E. (2011). Metabolism and tissue distribution of sulforaphane in Nrf2 knockout and wild-type mice. Pharm. Res..

[46] Daina A., Michielin O., Zoete V. (2017). SwissADME: A free web tool to evaluate pharmacokinetics, drug-likeness and medicinal chemistry friendliness of small molecules. Sci. Rep..

