# Supplementary figures and images for: Erucin, a Natural Isothiocyanate, Prevents Polyglutamine-Induced Toxicity in Caenorhabditis elegans via aak-2/AMPK and daf-16/FOXO Signaling
Source: Int J Mol Sci. 2024 Nov 14;25(22):12220. doi: 10.3390/ijms252212220 (PMC11594550; doi:10.3390/ijms252212220)

## Supplementary Table S1

In silico evaluation by SwissADME (<http://www.swissadme.ch/>)

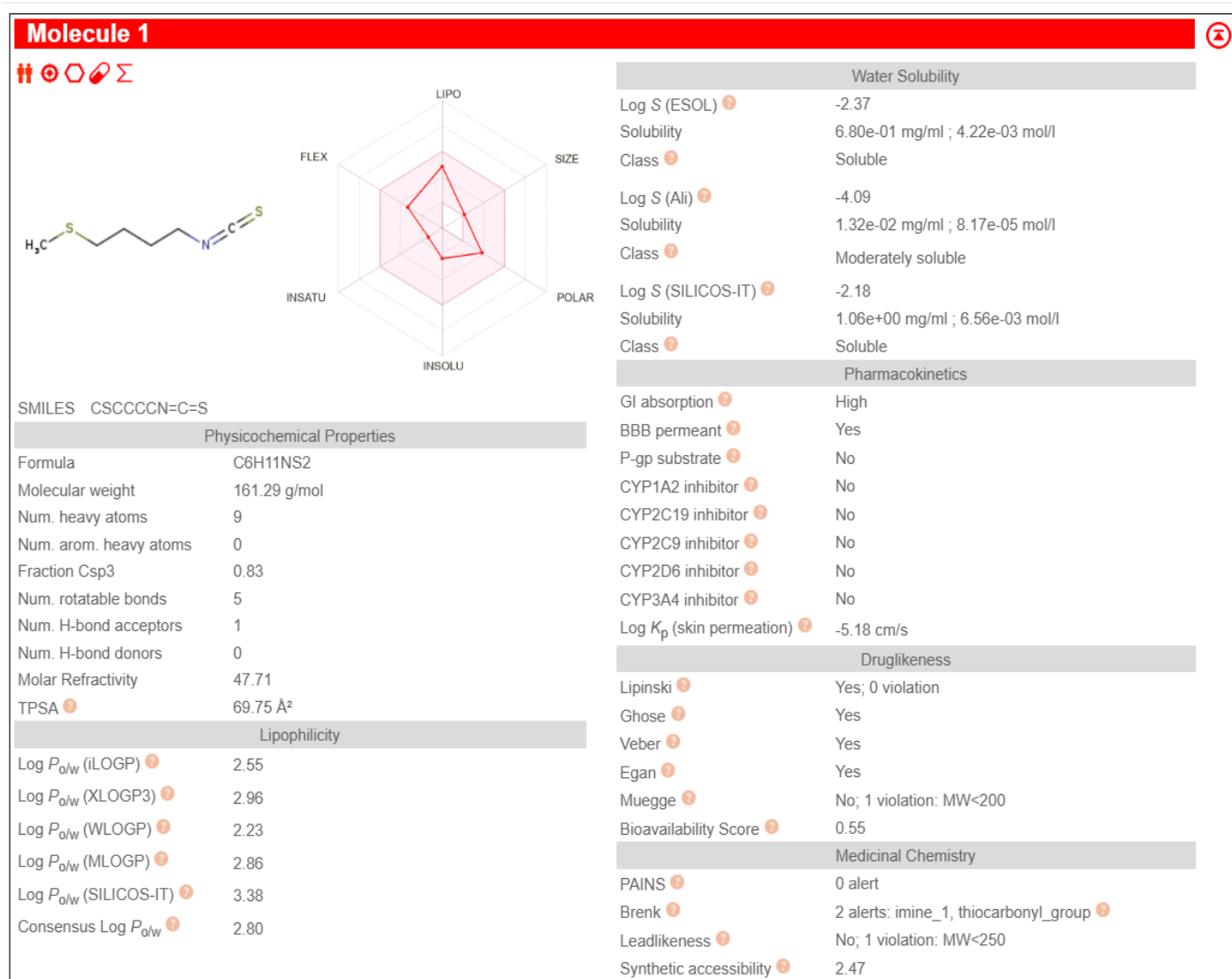

Supplement: Supplementary file 1 [file ijms-25-12220-s001.zip › ijms-3251925-supplementary.pdf]
